# Improving neurodevelopment in Zika-exposed children: A randomized controlled trial

**DOI:** 10.1371/journal.pntd.0010263

**Published:** 2022-03-08

**Authors:** Randall Waechter, Kemi S. Burgen, Bianca Punch, Roberta Evans, Karen Blackmon, Trevor Noël, Michelle Fernandes, Barbara Landon

**Affiliations:** 1 Department of Neuroscience, Physiology, and Behavioral Sciences; School of Medicine; St George’s University; Grenada, West Indies; 2 Windward Islands Research and Education Foundation; St. George’s University; Grenada, West Indies; 3 Caribbean Center for Child Neurodevelopment at WINDREF; Grenada, West Indies; 4 Mayo Clinic; Department of Psychiatry and Psychology; Jacksonville, Florida, United States of America; 5 Office of Research; St. George’s University; Grenada, West Indies; 6 Faculty of Medicine; Department of Paediatrics; University Hospitals Southampton; University of Southampton; Southampton, United Kingdom; 7 Nuffield Department of Women’s & Reproductive Health; John Radcliffe Hospital; University of Oxford; Oxford, United Kingdom; 8 School of Graduate Studies; St. George’s University; Grenada, West Indies; Baylor College of Medicine, UNITED STATES

## Abstract

**Background:**

While microcephaly is a significant adverse outcome of prenatal exposure to the Zika virus (ZIKV), subtle malformations of cortical development (MCD) have been observed in Zika-exposed children (ZEC), including delays in language, cognition, and motor domains, and visual acuity deficits. Interventions within the first 1,000 days of life can significantly improve developmental outcomes. This study examined a 12-week Responsive Caregiving Intervention on neurodevelopmental outcomes in 24-30-month-old ZEC.

**Methodology/Principal findings:**

A randomized controlled trial was implemented in Grenada, West Indies using an existing ZIKV cohort surveillance study. When children in that study turned 24 months, baseline child neurodevelopmental measures and caregiver interviews were administered. Caregivers who agreed to participate in the 12-week Responsive Caregiving Intervention, implemented when children were 24–30 months of age, were randomly assigned to the Intervention or Waitlist Control group. Children in both groups were re-assessed on the neurodevelopmental measures post-intervention.

**Conclusions/Significance:**

233 children from the ZIKV surveillance study met inclusion criteria, of which n = 80 declined participation, n = 42 did not complete the Intervention, and n = 72 missed follow-up assessments given strict timelines in the study design. The final sample for analysis was N = 13 children in the Intervention group and N = 26 children in the Control group. A GEE model analysis showed significantly higher language (p = 0.021) and positive behaviour (p = 0.005) scores for children in the Intervention group compared to the Control group. The Intervention had a medium effect on child language (d = 0.66) and a large effect on positive behaviour (d = 0.83). A 12-week Responsive Caregiving Intervention Programme significantly improves language and positive behaviour scores in 30-month-old normocephalic children who were exposed to ZIKV *in utero*. The programme provides an option for mothers of ZIKV-exposed children who are seeking an evidence-based neurodevelopmental intervention regardless of known impact of the virus on cortical formation.

**Trial registration:**

The study was registered with clinicaltrials.gov (NCT04697147).

## Author summary

The relationship between Zika virus exposure during pregnancy and microcephaly (abnormally small head size) in infants is well known. Prenatal Zika virus exposure can also cause more subtle impacts in infant language, cognition, and motor function, and visual acuity, even in the absence of microcephaly. Interventions in the first 1,000 days of life can significantly improve these outcomes in at-risk children. Few studies have been carried out with the aim of improving neurodevelopment in Zika-exposed children. The present study utilized a randomized controlled trial to examine the impact of a 12-week Intervention on neurodevelopmental outcomes in 24-30-month-old Zika-exposed children. Analysis showed significantly higher language and positive behaviour scores among children in the Intervention group compared to children in the Control group. The Intervention used in this study is relatively low-cost and easy to implement. The application of interventions from the global early child development field has implications for governments, policy makers, public health officials, and parents of Zika-exposed children who are seeking effective interventions to mitigate the risk of neurodevelopment delays in those children. The fact that these much-needed interventions already exist and can be rolled out relatively quickly and effectively is encouraging.

## Introduction

On 1 February 2016, the World Health Organization (WHO) declared a Public Health Emergency of International Concern (PHEIC) regarding microcephaly and other neurological disorders associated with the Zika virus (ZIKV). While the PHEIC was lifted on 18 November 2016, the WHO still considers the Zika epidemic and the associated complications a significant public health challenge requiring intense action, [1], and a global consortium has been established to examine pooled data from ZIKV infection [2].

Zika is a vector-borne virus spread via the bite of an infected *Aedes spp*. mosquito. It was first identified in Uganda in monkeys in 1947 and later in humans in 1952 [3]. The first recorded major ZIKV outbreak occurred in 2007 in the Federated States of Micronesia [3]. In 2015, the virus was identified in the Americas for the first time in Brazil and since then, it has spread to the rest of the region, including the Caribbean [4]. An outbreak of ZIKV was identified in the Caribbean Island of Grenada from April to November 2016 [5]. While existing data suggests that no cases have been reported in the Caribbean region since December 2016 [6], studies continue to examine the potential impact of infection on children born during the 2016 outbreak under the auspices of the original PHEIC.

The first evidence for a negative impact of prenatal ZIKV exposure on neurodevelopment came during the ZIKV outbreak in Brazil when an unusually high proportion of children were born with microcephaly. The presence of microcephaly in ZIKV-exposed children (ZEC) is a clear risk factor for adverse long-term outcomes such as developmental delays and seizures [7]. While microcephaly is a significant and obvious adverse outcome, more subtle malformations of cortical development (MCD) have also been observed in ZEC [8–10]. Even subtle MCDs, when strategically located, can contribute to cognitive deficits, intellectual disabilities, and learning disorders later in life [11,12]. Normocephalic ZEC can show delays in language, cognition, and motor domains [9,13–20]. In some normocephalic ZEC, visual acuity deficits are present, in the absence of any other cognitive, motor, language, or behavioural delays [21].

Interventions within the first 1,000 days of life can significantly improve developmental outcomes during early childhood with effects persisting across the life course (e.g., [22–25]). These interventions are the culmination of decades of global ECD work that initially focused on reducing child mortality and improving maternal health, as outlined by the United Nations (UN) Millennium Declaration [26], and shifted to improving child brain developmental outcomes via nurturing care [27] following a 53% decline in the under-5 mortality rate from 2000 to 2015 [28]. The nurturing care framework includes five components to maximize ECD outcomes: (1) good health; (2) adequate nutrition; (3) responsive caregiving; (4) safety and security; and (5) opportunities for early learning [29]. The nurturing care approach requires a multi-sectoral, collaborative effort that includes public policies, programmes, and services to support parents, caregivers, and communities in improving child development. A series on advancing ECD highlights the importance of interventions that support positive behaviour change among parents to achieve these nurturing care standards. These interventions, which are designed to augment the positive impact of basic health and nutrition, education, and protection interventions on ECD, reflect the Responsive Caregiving component, which is the parent or caregiver’s ability to notice, understand, and respond to the needs of his/her child appropriately [29].

While no studies with the specific aim of improving neurodevelopment among ZEC have been carried out in the Caribbean region, other interventions have shown improved outcomes in children who may be at risk for developmental delay in the absence of exposure to ZIKV. One such study is the Jamaican-developed Reach Up Intervention, a home visiting programme that supports mothers’ engagement with their children, thus promoting their children’s development [30]. The effectiveness of the Reach Up Programme has been measured in varying populations of at-risk children: low-socioeconomic status, severely malnourished, low birth weight, and stunted [30]. Studies have consistently demonstrated improvements in cognitive function and language or overall mental development among children who were exposed to the intervention [30]. In one study, Hamadani and colleagues [31] adapted the Reach Up Intervention for children at high risk of developmental delay in Bangladesh and found post-intervention improvements in children’s neurodevelopment [30,31].

The Reach Up Intervention involves parental education about early child brain development and techniques for child stimulation to improve developmental outcomes [30,31]. The Saving Brains Grenada Programme is another Responsive Caregiving Intervention that focuses on working with parents and caregivers to promote and teach self-regulation, social-emotional connection, and development. Waechter and colleagues [32] examined the impact of the intervention on 24-month-old children whose parents received the community-based training and noted that children whose caregivers were assigned to the Intervention group attained significantly higher post-intervention scores on measures of cognition, fine and gross motor skills, and language at 24 months of age compared to their peers who were assigned to the Waitlist Control group. Further, the intervention contributed the greatest level of variance among all measured ECD factors for fine motor, gross motor, and language development scores; and the second most variance for cognition scores [32].

Given the emerging evidence about the effectiveness of intervention programmes to improve ECD in low-and-middle-income countries and concerns about the potential subtle neurological impact of ZIKV on child development that can manifest as deficits in cognition, intellectual disabilities and learning disorders later in life, more evidence-based interventions are needed to determine whether neurodevelopment can be improved in ZEC. The present study aimed to examine the impact of a 12-week Responsive Caregiving Intervention on neurodevelopmental outcomes in 24-30-month-old ZEC. We hypothesized that ZEC whose parents received the Responsive Caregiving Intervention would show better neurodevelopmental outcomes at 2.5-year follow-up than those who were allocated to the Waitlist Control group.

## Methods

### Ethics statement

This study was approved by the St. George’s University Institutional Review Board (#16061). Written informed consent was obtained from all participants. The study was registered with clinicaltrials.gov (NCT04697147).

### Study design

A randomized controlled trial design was implemented to evaluate the impact of a Responsive Caregiving Intervention on children between the ages of 24–30 months in Grenada, West Indies. The sample was derived from an existing ZIKV cohort study in Grenada (see Blackmon et al. [21]). When the children in this existing surveillance study turned 24 months, baseline neurodevelopmental measures and parent/caregiver interviews were administered at the community health clinic closest to the participant. Once this baseline data was collected, the caregiver was asked if he/she would like to participate in the 12-week Intervention. Those who agreed to participate in the study were randomly assigned to the Intervention, or the Waitlist Control group. The intervention was implemented when the children were between 24 and 30 months of age while the Control group received no intervention. Children were then re-assessed on the neurodevelopmental measures post-intervention.

### Participants

All participants in the trial belonged to an ongoing ZIKV cohort study in Grenada, West Indies, with details regarding serum testing and ZIKV status for mothers and infants previously described (see Blackmon et al. [21]). For this trial, participants were recruited for the intervention when the child received his/her 24-month follow-up assessment for the ZIKV cohort study. Inclusion criteria consisted of: (i) active enrollment in the existing ZIKV cohort, (ii) child ≤ 24 months of age at the time of intervention enrollment, and (iii) child classified as ZIKV-exposed (see Blackmon et al. [21]).

Written consent and contact information were obtained from participants who wished to be involved and they were randomized to either Intervention or Control via random numbers table. Participants were contacted by a community health worker (Roving Caregiver) to schedule convenient days/times to complete their intervention sessions. A total of n = 233 parents/caregivers were contacted about participation in the study when their child turned 24-months old and n = 153 agreed and were randomized to either the Intervention or Control group. The intervention was provided to families between 24–29 months of the child’s life.

### Responsive caregiving intervention

The Responsive Caregiving Intervention used in the current study draws upon the US-based Conscious Discipline (CD) Programme, which has been adapted for and used in the study site (Grenada, West Indies) for more than 10 years. CD is a brain-based, trauma informed curriculum emphasizing the importance of social-emotional learning and self-regulation for fostering neurodevelopment in children through behavioural and perceptual change in adults. CD focuses on building a strong social-emotional connection between the child and his/her caregivers, both in home and in school environments. For this intervention, community health workers (called Roving Caregivers) were trained in the CD curriculum and visited the homes of the participants over the course of 12 weeks, for a 1-hour session each week, to work with caregiver-child dyads. Both caregiver and child were required to be present for each session. The Roving Caregiver Programme was established in 1992 to deliver stimulation through home visits to at-risk children [33]. In 2014 the Roving Caregivers adopted CD, which focuses on building caregiver-child social-emotional connection rather than stimulation for infants alone. The CD curriculum teaches seven skills associated with caregiver self-regulation and optimal responsive caregiving: Composure, Assertiveness, Encouragement, Choices, Empathy, Positive Intent, and Consequences [34]. In each week’s lesson, the Roving Caregivers demonstrated interactions with children and allowed parents/caregivers to practice these same activities—enjoyable songs and games—to build attunement through safe, predictable interactions using the variables required for connection: eye contact, touch, presence in the moment and sense of play. Caregivers were shown a simplified triune brain model that provides a rationale for safety and connection by describing brain states. More information on this CD-based Responsive Caregiving Intervention is available from the corresponding author who can provide a copy of the Manual for this 12-week Intervention.

The Control group was not exposed to the Responsive Caregiving Intervention. All ZEC in the Control group were scheduled for neurodevelopmental assessments at 24, 27, and 30 months of age to compare their outcomes to the Responsive Caregiving Intervention group.

### Outcome measures

Neurodevelopmental outcomes at 2 years were measured via the INTERGROWTH-21^st^ Neurodevelopment Assessment (INTER-NDA) [35,36]. The INTER-NDA is a multi-dimensional, standardized assessment measuring cognition, fine motor, gross motor, language, and positive and negative behaviour outcomes in children aged 22 to 30 months [35]. It was developed for and has been implemented in low-, middle-, and high-income populations [35]. Its 37 items are scored on a 5-point scale, characterizing the child’s performance across a spectrum rather than on the binary (pass/fail) scoring classification. The assessment combines the psychometric methods of direct assessments, reporters’ observation, and caregiver reports. The INTER-NDA takes approximately 20 minutes to complete and can be administered in the field by non-specialists. The INTER-NDA has previously been shown to have good agreement with the BSID III edition (intraclass correlation coefficients between 0.745 and 0.883 [p < 0.001] for all subscales) [36]; a test-retest reliability of k = 50.79 (95% CI: 0.48–0.96) and an interrater reliability of k = 50.70 (95% CI: 0.47–0.88) [35]. The INTER-NDA’s normative ranges for the standardized scores for each of its six subscales are international standards (as opposed to references) of child development and were developed according to the WHO’s prescriptive approach for the construction of a biological standard as applied in the WHO Multicentre Growth Reference Study [37]. It has been piloted and culturally customized for the Grenadian population and has been used in previous studies assessing neurodevelopment in Grenadian children [38]. The INTER-NDA was administered by research assistants who were masked to the child’s ZIKV status and group (i.e., Intervention versus Control).

Parent demographics and social-environmental assessment were measured via standardized surveys and questions that evaluated environmental conditions that could potentially affect the child’s development. These surveys included demographic and socioeconomic status (SES) questions such as primary caregiver, monthly income and number of persons living in the household, and pre- and post-natal behaviours of the mother. Standardized questionnaires included USDA Food Security Questionnaire [39,40], General Health Questionnaire (GHQ-12) [41], Social Support Questionnaire (adapted from Assessment of Parental Well-being and Behaviours) [42], Confusion, Hubbub and Order Scale (CHAOS) [43], and the Home Observation for Measurement of the Environment (HOME) [44]. The parent/caregiver interview was administered by a research assistant at the 24-month visit and took approximately 30 minutes to complete.

### Statistical analysis

All data were analyzed using the Statistical Package for the Social Sciences (SPSS) v. 26 (IBM Corp). Mean scores on the outcome measure (INTER-NDA) were converted to standardized scores per the procedure outlined by Fernandes and colleagues [37] and analyzed in SPSS.

T-tests and chi-square analyses were used to compare baseline socio-demographics between Intervention and Control groups. These tests were also used to compare baseline data for those participants who remained in the study and those lost to follow-up. Generalized estimating equation (GEE) [45] analyses were used to examine differences in child neurodevelopment (INTER-NDA scores) between Intervention and Control groups. To investigate the impact of the intervention, endpoint estimated marginal means derived from GEE models were used to calculate standardized effect sizes (Cohen’s d). Cohen’s d values were interpreted using the following reference values: 0.2, 0.5, 0.8 for small, medium, and large, respectively [46].

## Results

**Fig 1:**
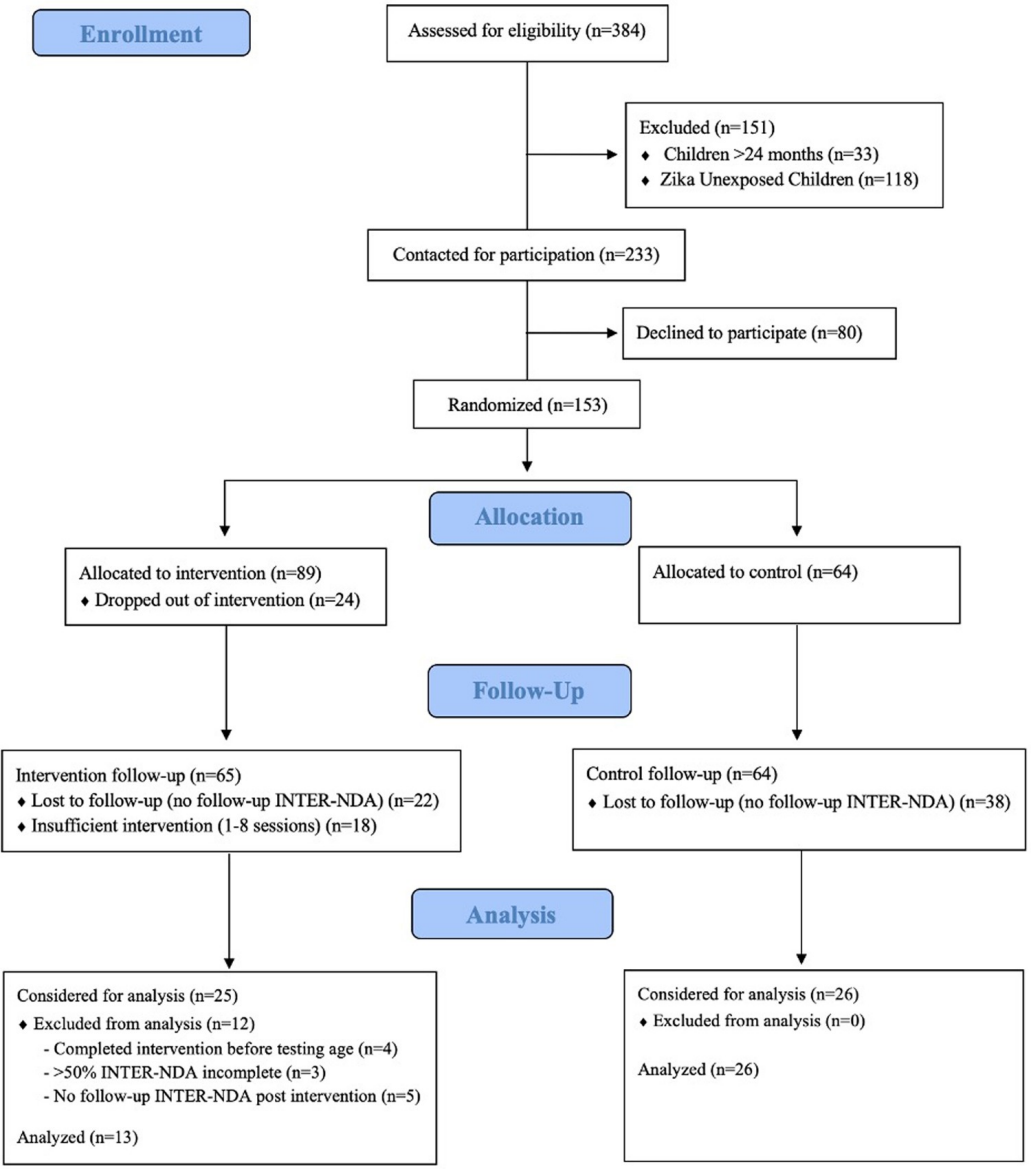
CONSORT Flow Diagram.

Caregivers of children enrolled in a pre-existing longitudinal ZIKV cohort study (N = 384) were eligible to participate in the CD-based Responsive Caregiving Intervention when their child turned 24 months of age (Fig 1). A total of n = 118 of those children were not exposed to ZIKV *in utero* and did not meet inclusion criteria. Additionally, n = 33 children were older than 24 months of age when the intervention study was initiated and were excluded. The remaining n = 233 children met inclusion criteria and their caregivers were approached about participating in the intervention when the child turned 24 months of age. A total of n = 80 (34%) declined participation, leaving n = 153 to be randomly assigned to the Intervention group (n = 89) or Control group (n = 64). Of the n = 89 participants assigned to the Intervention group, n = 24 dropped out due to scheduling conflicts or other reasons. A further n = 18 were removed from the Intervention group for completing fewer than 75% of the intervention classes (i.e., 9 of 12 sessions). Finally, n = 60 children across both groups (Intervention n = 22; Control n = 38) missed follow-up assessment sessions, resulting in a lack of neurodevelopmental scores. The remaining n = 51 participants (Intervention n = 25; Control n = 26) were considered for analysis. After data cleaning, n = 12 Intervention participants were excluded from the final analysis due to missing INTER-NDA scores (in these cases, item scores were not collected or were considered invalid due to distractions or child fatigue). No exclusions were made in the Control group. The final sample size for analysis included n = 13 children in the Intervention group and n = 26 children in the Control group (Fig 1).

Socio-demographic data collected at baseline for Intervention (n = 13) and Control (n = 26) groups were examined for any significant disparities between the groups. Results showed that the Intervention and Control groups were equivalent across all variables except parental education level (Table 1). A higher level of education was attained by parents in the Control group compared to the Intervention group (p < .001). Further analyses were run with and without parent education level as a covariate. Inclusion of this variable did not significantly change the results. Thus, the results reported below do not include parental education level as a covariate.

**Table 1:** Baseline Characteristics of the Sample.

**Sample Characteristics**	**Intervention (N = 13) M (SD)**	**Control (N = 26) M (SD)**	**Total (N = 39) M (SD)**	***p***
**Infant Age**	23.7 (0.59)	23.8 (0.67)	23.8 (0.64)	0.730
**Maternal General Health** ^**a**^	17.2 (3.56)	19.1 (6.02)	18.5 (5.36)	0.211
**Social Support** ^**b**^	39.7 (8.23)	33.6 (10.56)	35.6 (10.17)	0.074
**CHAOS** ^**c**^	26.7 (6.63)	26.8 (9.71)	26.8 (8.71)	0.970
**HOME** ^**d**^	35.6 (3.60)	31.8 (8.24)	33.1 (7.21)	0.061
**Sample Characteristics**	**Intervention (N = 13) N (%)**	**Control (N = 26) N (%)**	**Total (N = 39) N (%)**	***p***
**Infant Gender**				
Female	7 (54%)	15 (58%)	22 (56%)	0.819
Male	6 (46%)	11 (42%)	17 (44%)	
**Parent Age**				
18–30	9 (69%)	14 (54%)	23 (59%)	0.618
31–40	3 (23%)	10 (38%)	13 (33%)	
41–50	1 (8%)	2 (8%)	3 (8%)	
**Parent Education Level**				
Primary	0 (0%)	9 (35%)	9 (23%)	0.001
Secondary	10 (77%)	4 (15%)	14 (36%)	
Tertiary	3 (23%)	13 (50%)	16 (41%)	
**Monthly Income**				
< $500 XCD	1 (8%)	1 (4%)	2 (5%)	0.655
$500–1000 XCD	2 (15%)	4 (15%)	6 (15%)	
$1001–2000 XCD	5 (38%)	7 (27%)	12 (31%)	
$2001–3000 XCD	0 (0%)	2 (8%)	2 (5%)	
$3001+ XCD	2 (15%)	8 (30%)	10 (26%)	
**Marital Status**				
Single	6 (46%)	8 (30%)	14 (36%)	0.611
Domestic Partnership	4 (31%)	9 (35%)	13 (33%)	
Married	3 (23%)	9 (35%)	12 (31%)	
**Birth Complications**				
Yes	3 (23%)	5 (19%)	8 (21%)	0.729
No	9 (69%)	20 (77%)	29 (74%)	
**Complications Post Birth**				
Yes	5 (38%)	6 (23%)	11 (28%)	0.351
No	8 (62%)	19 (70%)	27 (69%)	
**Feeding**				
Breastfed	6 (46%)	8 (30%)	14 (37%)	0.436
Bottle-fed	2 (15%)	2 (8%)	4 (10%)	
Both	5 (38%)	15 (58%)	20 (51%)	
**Food Security**				
Food Secure	8 (62%)	14 (54%)	22 (56%)	0.843
Food Insecure (Moderate)	2 (15%)	6 (23%)	8 (21%)	
Food Insecure (Severe)	3 (23%)	6 (23%)	9 (23%)	

^a^ Higher scores on the GHQ-12 indicate worse mental health

^b^ Higher scores on the SSQ indicate more social support

^c^ Higher scores on the CHAOS indicate a more chaotic home environment

^d^ Higher scores on the HOME indicate a better home environment

An analysis to determine the equivalence of the participants who remained in the study to those who were lost to follow-up in both the Intervention and Control groups was run on baseline data. The results indicated that those lost to follow-up were equivalent to those who remained in the Control group of the study, whereas the Intervention group showed some disparity on child age and monthly income. Those lost to follow-up (M = 24.39) in the Intervention group showed a higher child age than those who remained (M = 24.00, p = 0.027); and those lost to follow-up in the Intervention group showed higher monthly incomes than those who remained (p = 0.041) (S1 Table). Child age and monthly income were included as covariates in the main analysis comparing child neurodevelopment scores between the Control and Intervention groups to ensure that these variables were not driving the outcomes. Neither variable significantly contributed to neurodevelopmental outcomes when comparing the Intervention and Control groups.

**Fig 2:**
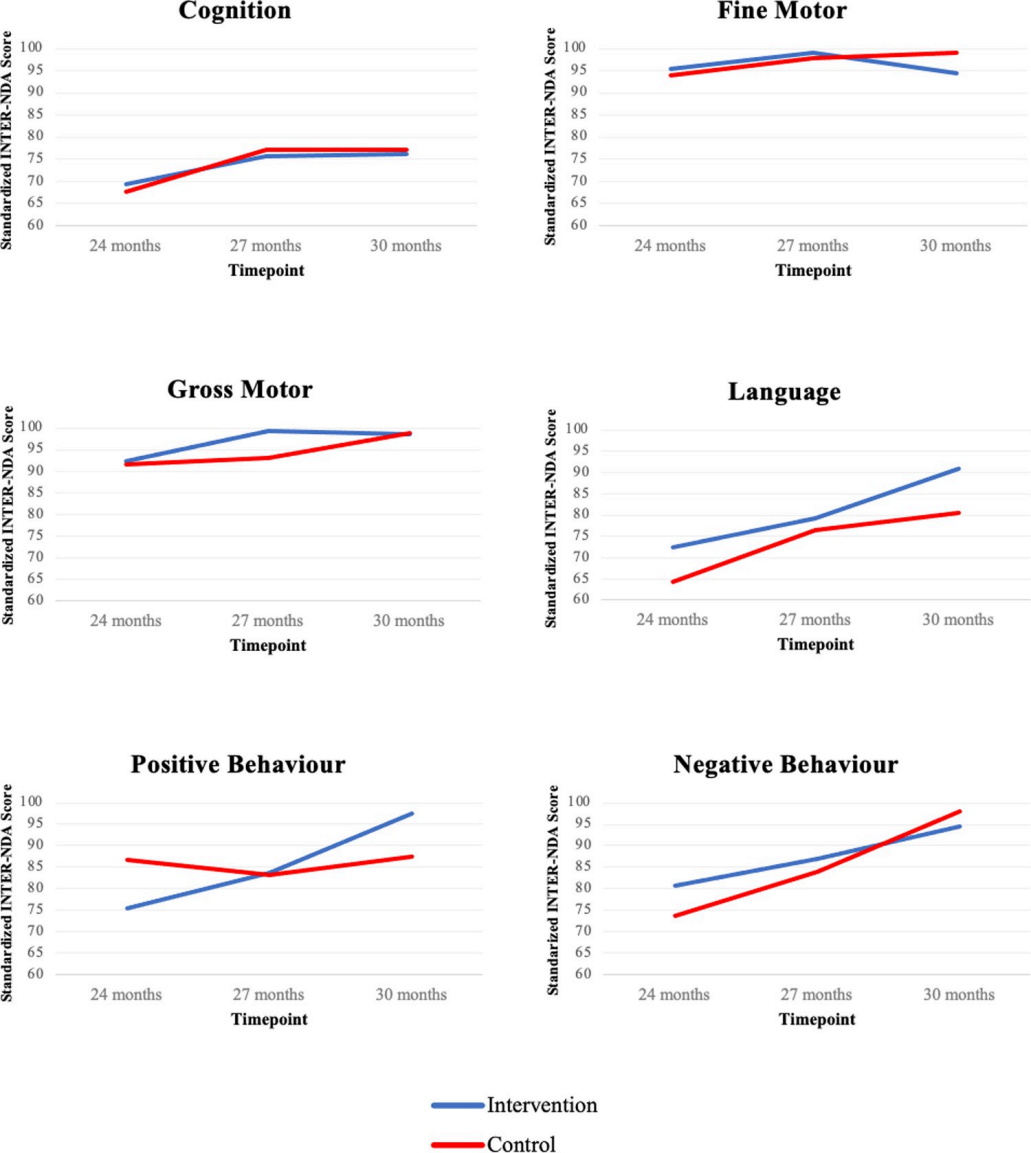
Fitted GEE Model Plots of Child Development Domains Over Time by Group.

Results of a GEE model analysis showed significantly higher language (p = 0.021) and positive behaviour (p = 0.005) standardized scores on the INTER-NDA for the children in the CD-based Responsive Caregiving Intervention group compared to the children in the Control group (Table 2). The intervention had a medium effect on child language (d = 0.66) and a large effect on positive behaviour (d = 0.83) (Table 3). Both groups scored similarly on the remaining four domains of the INTER-NDA (cognition, gross motor, fine motor, negative behaviour) (Table 3 and Fig 2).

**Table 2:** Intervention Effects on Child Development Domains Measured by the INTER-NDA.

INTER-NDA Domain	B	CI Lower	CI Upper	*p*
Cognition	-1.0	-8.7	6.7	0.799
Fine Motor	-4.7	-11.2	1.8	0.153
Gross Motor	-0.3	2.5	-5.3	0.891
Language	10.5	1.6	19.3	0.021
Positive Behaviour	9.9	3.0	16.8	0.005
Negative Behaviour	-3.4	-12.2	5.5	0.453

**Table 3:** Estimated Marginal Means of Child Development Outcomes Derived from the GEE Models by Group and Effect Sizes.

INTER-NDA Subscale	Time	Group	M	CI Lower	CI Upper	*d*
Cognition	1 (24 months)	Intervention (N = 13)	69.3	61.4	77.3	
		Control (N = 26)	67.7	63.4	71.9	
	2 (27 months)	Intervention (N = 13)	75.8	69.5	82.0	
		Control (N = 26)	77.1	72.5	81.7	
	3 (30 months)	Intervention (N = 13)	76.2	70.0	82.3	0.085
		Control (N = 26)	77.2	72.6	81.8	
Fine Motor	1 (24 months)	Intervention (N = 13)	95.5	90.9	100.1	
		Control (N = 26)	93.9	90.0	97.8	
	2 (27 months)	Intervention (N = 13)	99.2	97.6	100.7	
		Control (N = 26)	97.7	95.4	100.1	
	3 (30 months)	Intervention (N = 13)	94.4	88.1	100.7	0.544
		Control (N = 26)	99.1	97.6	100.7	
Gross Motor	1 (24 months)	Intervention (N = 13)	92.3	87.3	97.3	
		Control (N = 26)	91.5	86.8	96.1	
	2 (27 months)	Intervention (N = 13)	99.3	97.9	100.7	
		Control (N = 26)	93.1	89.2	96.9	
	3 (30 months)	Intervention (N = 13)	98.6	95.5	101.6	0.042
		Control (N = 26)	98.9	95.0	102.8	
Language	1 (24 months)	Intervention (N = 13)	72.3	63.7	80.8	
		Control (N = 26)	64.2	55.4	73.0	
	2 (27 months)	Intervention (N = 13)	79.2	71.4	86.9	
		Control (N = 26)	76.5	69.1	83.9	
	3 (30 months)	Intervention (N = 13)	90.9	88.0	93.9	0.659
		Control (N = 26)	80.5	72.1	88.8	
Positive Behaviour	1 (24 months)	Intervention (N = 13)	75.4	68.8	82.0	
		Control (N = 26)	86.8	79.8	93.7	
	2 (27 months)	Intervention (N = 13)	83.7	76.6	90.7	
		Control (N = 26)	83.1	76.6	89.6	
	3 (30 months)	Intervention (N = 13)	97.5	94.2	100.8	0.833
		Control (N = 26)	87.6	81.5	93.6	
Negative Behaviour	1 (24 months)	Intervention (N = 13)	80.8	67.5	94.0	
		Control (N = 26)	73.7	62.7	84.7	
	2 (27 months)	Intervention (N = 13)	87.1	73.6	100.6	
		Control (N = 26)	83.9	75.4	92.4	
	3 (30 months)	Intervention (N = 13)	94.5	88.8	100.3	0.234
		Control (N = 26)	97.9	91.2	104.6	

## Discussion

We have shown that a 12-week Responsive Caregiving Intervention programme significantly improves language and positive behaviour scores in 30-month-old normocephalic children who were exposed to ZIKV *in utero*. The findings of our study are consistent with previous reports investigating the impact of home-visiting early childhood interventions including those involving children born to low-income families [47–54], children with or at risk of developmental delays [30,31,48,50,52,53,55–58], and children with other health conditions [50,55]. Our findings are also consistent with a Brazilian study designed to improve developmental outcomes in children with congenital Zika syndrome (CZS) by using the principles of the Goals-Activity-Motor Enrichment (GAME) based Programme. GAME is an intervention that involves: (1) goal-oriented motor training; (2) parental education; and (3) enrichment of the child’s motor learning environment [59]. The 16-week pilot study, which involved infants with CZS between the ages of 3 and 9 months, examined the effect of the GAME Programme on how mothers perceived their children’s achievement of functional goals, (e.g., rolling during play, holding toys, sitting to play, or be fed, and maintaining head/trunk control while being carried) and whether the Programme improved infants’ cognitive and motor abilities [59]. The study further examined whether the GAME protocol would result in enrichment in the child’s home environment and how the mothers perceived the service they were given [59]. A total of 22 infants in the study received the GAME Programme while 10 were in the control group and received traditional care that did not involve active participation by the mother [59]. The results of the study showed an improvement in mothers’ rating of infants’ performance in functional goals and enrichment of the home environment for those that received the GAME Intervention [59]. Neither the GAME group nor the Control group showed any significant improvements in cognitive and motor function [59]. No other known studies have examined the impact of an intervention on neurodevelopmental outcomes specifically in ZEC with and without microcephaly. Our study is unique in that it is the first time an ECD-based Responsive Caregiving Intervention has been shown to improve neurodevelopmental outcomes in ZEC.

The results of this study are consistent with a recent CD-based Responsive Caregiving Intervention study carried out in Grenada, West Indies [32]. Waechter et al. [32] found significantly higher scores across the neurodevelopmental domains of cognition, fine motor, gross motor, and language in 24-month-old children exposed to the Responsive Caregiving Intervention versus children allocated to a Waitlist Control group. The intervention contributed the greatest level of variance among all measured ECD factors for fine motor scores (d = 0.524), gross motor scores (d = 0.238), and language development scores (d = 0.259); and the second most variance for cognition scores (d = 0.216). This previous study differed from the neurodevelopmental outcomes seen in ZEC randomly assigned to the Intervention group in the present study, who did not show improvement in cognition and fine or gross motor neurodevelopment scores. A potential explanation for this difference is that Waechter and colleagues [32] intervened with children prior to 24 months of age and assessed these children at the 24-month timepoint, whereas in the present study, children received the intervention between the ages of 24 and 30 months and were assessed at the 24-, 27-, and 30-month timepoints. This difference in age at the time of the intervention and assessment could account for the differences in the overall effect of the intervention as an earlier start age for intervention could predict a bigger overall effect on neurodevelopment across multiple domains [60].

The main limitations of the current study are its small final sample size and high levels of attrition between initial contact for inclusion in the study and drop out from the intervention. Moreover, some participants were excluded from analysis due to missing neurodevelopment data post-intervention. Study protocol adherence and lost follow-up are significant limitations that are challenging to overcome and are not uncommon in community-based intervention studies with longitudinal follow-ups [50]. Another significant limitation is the lack of blinding to group allocation among both the participants themselves, as well as the Roving Caregivers who provided the intervention. Just by knowing they were receiving an intervention, the parents may have interacted differently with their children, and that may have impacted neurodevelopmental outcomes in the children. There was also a chance of contamination between the Intervention and Control groups given the relatively small size of Grenada, though we believe this was unlikely given the small final sample size of the Intervention group, wide distribution of families across different villages, and the fact that most families do not travel far from their village. In an effort to counteract these weaknesses, we ensured that all personnel who assessed neurodevelopmental outcomes in the children were blinded to the group assignment of the children (i.e., Control vs. Intervention).

All the children in the present study were exposed to ZIKV *in utero*. While this allowed for a direct comparison of neurodevelopmental outcomes between those who received the Intervention and those in the Control group, it was not possible to determine whether any or all the ZEC in the study had experienced subtle MCDs and/or neurocognitive impairment and/or neurodevelopmental delay because of their exposure to ZIKV. Future studies would need to include ZEC with documented MCDs and/or neurocognitive impairment and/or neurodevelopmental delay to determine whether the intervention can improve neurodevelopmental outcomes in children with ZIKV-related impairment short of microcephaly.

The CD-based Responsive Caregiving Intervention used in the present study prioritizes the building of social-emotional connections between caregiver and child to improve child neurodevelopment. We hypothesize that the nature of this intervention lends itself to the increase in positive behaviour and language abilities seen in the children randomly assigned to the Intervention group. The intervention used in the present study includes several activities that help forge a relationship between caregiver and child. These activities include singing songs and rhymes, reading stories, allowing, and encouraging the child to express themselves verbally, and equipping children with tools for expressing certain needs and wishes. These activities likely explain the improved language scores amongst our Intervention group.

The CD-based Responsive Caregiving Intervention used in the present study and similar interventions can be low-cost and relatively easy to implement. This could lead to more children having access to interventions that can mitigate neurodevelopmental delays in cases of arboviral diseases or otherwise. The cross-translation of evidence-based interventions from the global ECD field has implications for governments, policy makers, public health officials, ECD experts and parents of ZEC who are seeking effective interventions to mitigate the risk of neurodevelopment delays in those children. The fact that these much-needed interventions already exist and can be rolled out relatively quickly and effectively is encouraging. Moreover, the diversity of ECD interventions allows for a selection of programmes that apply best to regional, cultural, and economic variance.

## Supporting information

S1 Table Group equivalence analysis of covariate baseline data, including standardized INTER-NDA scores (/100) between those who remained in the Intervention (N = 25) and Control groups (N = 26) vs. those lost to follow-up in the Intervention group (N = 64) and Control group (N = 38). (DOCX)
