# Supplementary material for: Improving neurodevelopment in Zika-exposed children: A randomized controlled trial
Source: PLoS Negl Trop Dis. 2022 Mar 8;16(3):e0010263. doi: 10.1371/journal.pntd.0010263 (PMC8903297; doi:10.1371/journal.pntd.0010263)
Supplement: S1 Table — (DOCX) [file pntd.0010263.s001.docx]

**S1 Table. Group equivalence analysis of covariate baseline data, including standardized INTER-NDA scores (/100) between those who remained in the Intervention (N=25) and Control groups (N=26) vs. those lost to follow-up in the Intervention group (N=64) and Control group (N=38).**

| **Group** | **Covariate** | **Remained N (%)** | **Lost to Follow-Up N (%)** | ***p*** |
| --- | --- | --- | --- | --- |
| Intervention | **Infant Gender** Female Male | 13 (59%) 9 (41%) | 21 (38%) 35 (62%) | 0.084 |
| Control |  | 8 (40%) 12 (60%) | 13 (39%) 20 (61%) | 0.965 |
| Intervention | **Parent Age** 18-30 31-40 41-50 | 12 (55%) 8 (36%) 2 (9%) | 35 (61%) 17 (30%) 5 (9%) | 0.866 |
| Control |  | 12 (60%) 8 (40%) 0 (0%) | 21 (60%) 12 (34%) 2 (6%) | 0.470 |
| Intervention | **Parent Education Level** Primary Secondary Tertiary | 4 (18%) 8 (37%) 10 (45%) | 8 (14%) 27 (46%) 23 (40%) | 0.700 |
| Control |  | 4 (19%) 7 (33%) 10 (48%) | 5 (14%) 22 (61%) 9 (25%) | 0.118 |
| Intervention | **Monthly Income** < $500 XCD $500-1000 XCD $1001-2000 XCD $2001-3000 XCD $3001+ XCD | 3 (15%) 5 (25%) 2 (10%) 2 (10%) 8 (40%) | 1 (2%) 11 (25%) 18 (40%) 6 (13%) 9 (20%) | 0.041 |
| Control |  | 0 (0%) 3 (19%) 4 (25%) 1 (6%) 8 (50%) | 0 (0%) 4 (12%) 14 (44%) 8 (25%) 6 (19%) | 0.077 |
| Intervention | **Marital Status** Single Domestic Partnership Married | 4 (18%) 9 (41%) 9 (41%) | 24 (41%) 23 (40%) 11 (19%) | 0.063 |
| Control |  | 13 (62%) 4 (19%) 4 (19%) | 17 (50%) 9 (26%) 8 (24%) | 0.740 |
| Intervention | **Birth Complications** Yes No | 3 (14%) 18 (86%) | 13 (23%) 43 (77%) | 0.390 |
| Control |  | 2 (10%) 19 (90%) | 9 (26%) 26 (74%) | 0.140 |
| Intervention | **Post Birth Complications** Yes No | 6 (28%) 16 (72%) | 17 (30%) 40 (70%) | 0.823 |
| Control |  | 8 (38%) 13 (62%) | 10 (28%) 26 (72%) | 0.419 |
| Intervention | **Feeding** Breastfed Bottle-fed Both | 10 (45%) 3 (14%) 9 (41%) | 18 (32%) 6 (11%) 32 (57%) | 0.430 |
| Control |  | 8 (38%) 2 (10%) 11 (52%) | 9 (26%) 2 (5%) 24 (69%) | 0.477 |
| Intervention | **Food Security** Food Secure Food Insecure (Moderate) Food Insecure (Severe) | 11 (55%) 3 (15%) 6 (30%) | 33 (57%) 12 (21%) 13 (22%) | 0.737 |
| Control |  | 14 (67%) 3 (14%) 4 (19%) | 16 (45%) 13 (36%) 7 (19%) | 0.174 |
| **Group** | **Covariate** | **Remained M (SD)** | **Lost to Follow-Up M (SD)** | ***p*** |
| Intervention | Child Age | 24.00 (.54) | 24.39 (.95) | 0.027 |
| Control |  | 24.35 (.87) | 24.06 (.49) | 0.187 |
| Intervention | Maternal General Health^a^ | 22.61 (6.80) | 23.38 (3.08) | 0.608 |
| Control |  | 22.29 (5.02) | 22.36 (3.44) | 0.952 |
| Intervention | Social Support^b^ | 36.27 (8.68) | 34.36 (11.56) | 0.482 |
| Control |  | 35.17 (8.11) | 36.14 (8.75) | 0.857 |
| Intervention | CHAOS^c^ | 38.68 (4.36) | 38.25 (4.87) | 0.715 |
| Control |  | 37.90 (4.58) | 37.31 (4.08) | 0.611 |
| Intervention | HOME^d^ | 34.73 (5.60) | 33.73 (6.28) | 0.519 |
| Control |  | 34.45 (6.37) | 32.17 (6.71) | 0.233 |
| **Group** | **INTER-NDA Subscale** | **Remained M (SD)** | **Lost to Follow-Up M (SD)** | ***p*** |
| Intervention | Cognition | 65.35 (16.83) | 67.49 (13.04) | 0.526 |
| Control |  | 66.68 (12.04) | 63.05 (14.21) | 0.309 |
| Intervention | Fine Motor | 95.22 (8.77) | 91.27 (11.91) | 0.136 |
| Control |  | 94.07 (10.19) | 96.02 (11.07) | 0.490 |
| Intervention | Gross Motor | 91.56 (11.24) | 93.55 (11.12) | 0.453 |
| Control |  | 92.42 (10.51) | 91.92 (12.54) | 0.871 |
| Intervention | Language | 60.97 (19.93) | 67.35 (20.11) | 0.184 |
| Control |  | 67.67 (20.71) | 64.51 (22.44) | 0.582 |
| Intervention | Positive Behaviour | 80.40 (19.47) | 77.09 (23.87) | 0.541 |
| Control |  | 83.18 (21.47) | 75.93 (22.63) | 0.218 |
| Intervention | Negative Behaviour | 70.00 (36.08) | 72.50 (31.45) | 0.750 |
| Control |  | 67.05 (35.68) | 58.72 (38.14) | 0.398 |

^a^ Higher scores on the GHQ-12 indicate worse mental health

^b^ Higher scores on the SSQ indicate more social support

^c^ Higher scores on the CHAOS indicate a more chaotic home environment
^d^ Higher scores on the HOME indicate a better home environment
